# Effect of Cooling Methods on CFRP–Concrete Bond Behavior After High-Temperature Exposure: An Experimental Study

**DOI:** 10.3390/polym18080939

**Published:** 2026-04-11

**Authors:** Bu Wang, Abdulmalik Al-barawi, Zhenxun Dai, Kehang Liu, Mostafa M. A. Mostafa, Mu Ma

**Affiliations:** 1 School of Civil Engineering, Chang’an University, Xi’an 710064, China; 2023128016@chd.edu.cn; 2 China Construction Science and Industry Corporation Ltd., Shenzhen 518054, China; 10114718@cscec.com; 3 Civil Engineering Department, Faculty of Engineering, Al-Azhar University, Qena 83513, Egypt; mostafa.mohamed@azhar.edu.eg; 4 China Northwest Architectural Design and Research Institute Co., Ltd., Xi’an 710018, China; mamu@cscec.com

**Keywords:** bond–slip, cooling methods, bond strength, CFRP sheet, fire-damaged concrete, double-lap shear test, statistics

## Abstract

Concrete structures are highly vulnerable to fire exposure, which accelerates the degradation of mechanical properties and may lead to partial or total structural failure. Externally bonded carbon fiber-reinforced polymer (CFRP) systems are widely used for post-fire strengthening; however, the bond behavior at the interfaces between CFRP and fire-damaged concrete, particularly under different cooling conditions, is not yet fully understood. In this study, the bond behavior was investigated experimentally and theoretically. Double-lap joint tests of thirty-nine specimens were conducted, including three unheated control specimens and thirty-six specimens exposed to temperatures of 200 °C, 400 °C, and 600 °C for durations of one and two hours. Two cooling methods, natural air cooling and water cooling, were applied prior to CFRP bonding. The results indicated that bond strength increased under exposure conditions of no more than 400 °C, whereas a significant reduction was observed at 600 °C. Water cooling resulted in lower bond strength compared with air cooling, while longer exposure durations improved bond performance under certain thermal conditions. The reasons behind the phenomena were analyzed in detail. Based on the experimental results, an analytical model for predicting the bond strength at the interfaces between fire-damaged concrete and CFRP sheets was developed. The model can account for the effects of peak temperatures, exposure durations, and cooling methods, and demonstrated high predictive accuracy (R^2^ = 0.94). The findings provide valuable insight into CFRP–concrete interaction after fire exposure and offer practical guidance for the assessment and rehabilitation of fire-damaged concrete structures.

## 1. Introduction

Concrete is the most widely used construction material globally due to its versatility and compressive strength [1,2,3,4,5]. However, its performance under extreme conditions, particularly fire, remains a critical concern for structural safety [6,7,8]. Fire exposure induces severe thermal stresses and chemical transformations in concrete [9], leading to phenomena such as explosive spalling, microcracking, and degradation of mechanical properties [10,11,12,13]. This deterioration compromises structural integrity, often resulting in catastrophic failure or requiring costly repairs [10,11,12,13,14,15,16,17,18,19,20,21,22]. Concurrently, fiber-reinforced polymer (FRP) composites, especially carbon FRP (CFRP), have emerged as an effective solution for strengthening and rehabilitating reinforced concrete (RC) structures [14,15,16,17,18,19]. CFRP offers several advantages, including a high strength-to-weight ratio, corrosion resistance, and ease of application [23,24,25,26,27,28,29], making it suitable for enhancing the load-bearing capacity of structural members such as beams, columns, and slabs [30,31,32,33,34,35].

Extensive research has been conducted on concrete fire performance and CFRP strengthening [36,37,38,39]. The fire resistance of concrete and the thermo-mechanical properties of CFRP materials have been widely studied [36,37,38,39,40,41,42,43]. In addition, the bond behavior between CFRP and concrete at ambient conditions has been thoroughly investigated, leading to the development of reliable strengthening models [43]. More recently, research has begun to focus on the interaction between these two aspects, particularly the bond performance of CFRP applied to fire-damaged concrete. Previous studies have demonstrated that elevated temperatures significantly degrade the CFRP–concrete bond, with bond strength reductions occurring at temperatures as low as 100 °C and becoming severe beyond 400–500 °C [44,45,46,47]. Other studies have examined the effects of CFRP configurations, insulation strategies, and mechanical anchorage systems on post-fire performance [48,49,50,51,52,53,54]. Additionally, some investigations have compared different cooling regimes, such as furnace cooling and water quenching, highlighting their influence on the post-heating condition of concrete [55,56,57,58,59,60,61,62,63]. It should be noted that most of these studies focus on bond behavior during elevated temperature exposure, whereas the present study investigates the residual bond performance after heating and subsequent cooling. In this study, the CFRP system is applied after the heating and cooling stages; therefore, the epoxy adhesive is not exposed to elevated temperatures during the thermal process. In addition to epoxy-based FRP systems, alternative strengthening approaches using inorganic matrices, such as fabric-reinforced cementitious matrix (FRCM), have been developed before to overcome the limitations of organic resins at elevated temperatures; these systems exhibit improved thermal stability and bond performance under high-temperature conditions [64]. Nevertheless, CFRP systems remain widely used in practice, which justifies the continued investigation of their residual bond behavior after fire exposure.

Despite these valuable contributions, most existing studies primarily focus on the influence of peak temperature and, to a lesser extent, exposure duration. Several works have provided important insights into bond degradation mechanisms under elevated temperatures. However, the post-fire cooling phase, which represents a critical stage in real fire scenarios, is often treated as a secondary or uncontrolled factor. In particular, the influence of different cooling methods (e.g., air cooling versus water quenching) on residual bond behavior remains insufficiently investigated. Moreover, existing analytical and predictive models rarely incorporate the cooling method as a governing parameter. Consequently, there is still a lack of comprehensive experimental data and modeling approaches that systematically evaluate and integrate the combined effects of temperature, exposure duration, and cooling method on CFRP–concrete bond performance.

To address this gap, the present study focuses on the residual bond behavior after thermal exposure and subsequent cooling and conducts a systematic experimental and analytical investigation of CFRP–concrete interfaces. A series of double-lap shear tests are performed on specimens subjected to controlled thermal conditions, in which peak temperature, exposure duration, and cooling method are independently varied. Based on experimental results, a predictive model is developed to estimate the residual bond strength, explicitly incorporating the cooling method alongside temperature and exposure duration. The proposed model is validated through statistical analysis, including the coefficient of variation (COV), *t*-test, and *p*-value evaluation. This study provides a more comprehensive and practically applicable framework for assessing and rehabilitating fire-damaged concrete structures strengthened with CFRP.

## 2. Materials and Methods

### 2.1. Details of Specimens

The experimental program has been conducted, consisting of 39 concrete prism samples, each measuring 150 × 150 × 200 mm, to evaluate bond strength. In addition, cubes with dimensions of 100 × 100 × 100 mm were cast in equal quantities and tested under the same heating and cooling conditions to test their residual compressive strengths. Strength losses resulting from various heating and cooling procedures were evaluated by comparing the overall strength of these samples with that of the control cubes. Both sets of samples were cast and air-cured for 24 h before being placed in a central curing room for 28 days. Table 1 shows the experimental program details. The specimens were divided into four groups. The first group, composed of three samples, served as the control group and was not exposed to heat. The remaining three groups were exposed to specified temperatures using an electric thermal oven with a maximum temperature capacity of 1000 °C, as shown in Table 1. The manuscript proposed a method to provide highlights towards the engineering applications within the scope. Furthermore, the experimental results provide practical guidance and a predictive tool for engineers to answer the critical question: What bond strength can be achieved when repairing this fire-damaged concrete with CFRP?

#### 2.1.1. Compressive Strength Test of Concrete

The concrete mixture was designed to achieve a compressive strength of 35 MPa, with a w/c ratio of 0.39. The mixture consisted of 330 kg/m^3^ of cement, 742 kg/m^3^ of sand, 1088 kg/m^3^ of gravel, 70 kg/m^3^ of fly ash, and 14.5 kg/m^3^ of superplasticizer additives. Antifreeze material was added into the mixture to counteract the effects of cold weather during pouring. The concrete mixture was poured into preprepared moulds measuring 150 × 150 × 200 mm for bond testing and 100 × 100 × 100 mm for compressive strength testing. Subsequently, the samples were placed in the central curing room for 28 days. The concrete compression material test was conducted in accordance with GB/T50081-2019 [65]. Table 2 shows the test results for compressive strength, including the sample count, average values, and standard deviation (SD).

#### 2.1.2. Tensile Test of FRP Sheets

The tensile test of CFRP sheets was conducted in accordance with ASTM standards [66,67] to determine the ultimate tensile strength, elastic modulus, and failure modes. Five specimens were prepared for each test, satisfying the minimum requirement specified by the standards. The CFRP coupons, with nominal dimensions of 250 × 25 mm, were fabricated using the hand lay-up technique and equipped with E-glass fiber tabs at both ends to prevent premature gripping failure.

Unidirectional TORAYCA UT70-30 CFRP sheets (Toray Co., Ltd., Tokyo, Japan) and a two-component epoxy resin (Konishi E2500S, Konishi Co., Ltd., Osaka, Japan) were used to prepare the specimens. The epoxy resin has a typical glass transition temperature (T_g_) in the range of 60–80 °C, depending on its formulation. The CFRP sheets had a unidirectional fiber density of 300 g/m^2^, a nominal width of 500 mm, and a nominal dry fiber thickness of 0.167 mm per layer.

The laminates were fabricated in accordance with ASTM D7565 [67] using the hand lay-up technique, in which the dry fiber sheets were impregnated with epoxy resin mixed at a ratio of 2:1 (resin:hardener). The layers were successively saturated and stacked to form a flat laminate, which was then cured under ambient conditions. Due to the hand lay-up process, the final laminate thickness reached approximately 1.0 mm, reflecting the combined contribution of fiber and epoxy as well as the presence of resin-rich regions.

It should be noted that the measured laminate thickness includes both fiber and epoxy contributions, whereas the nominal thickness corresponds only to the dry fiber sheet. As reported in previous studies, the laminate thickness depends on fiber thickness, epoxy content, and fiber volume fraction, and may exceed the nominal fiber thickness due to fabrication variability [68]. To ensure consistency and minimize the influence of thickness variability, the tensile stress was calculated based on the nominal fiber thickness rather than the measured laminate thickness.

**Figure 1:**
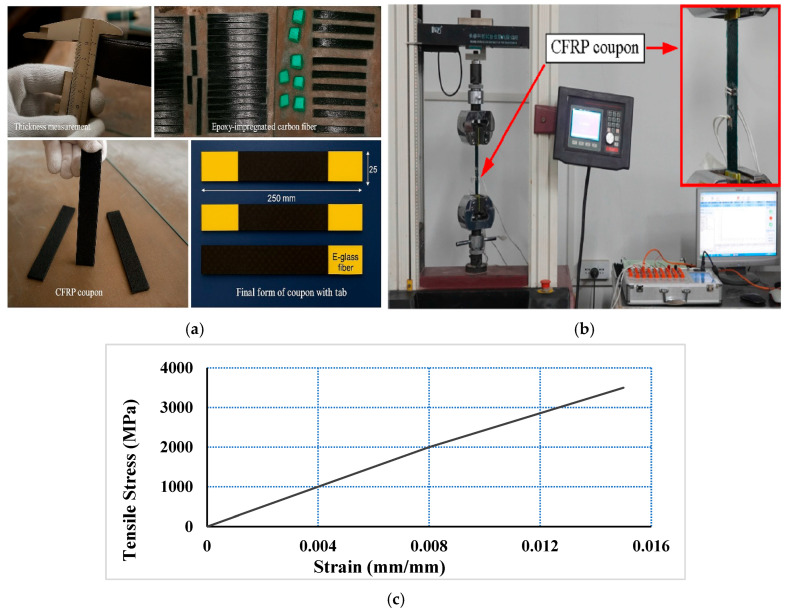
Tensile testing of CFRP coupons: (**a**,**b**) Tensile testing setup; and (**c**) Typical tensile stress–strain curve with one layer thick.

The tests were conducted under displacement control at a loading rate of 2 mm/min. The specimens exhibited linear elastic behavior up to failure, with fiber rupture and localized delamination as the dominant failure modes, as shown in Figure 1. The measured tensile properties are summarized in Table 3.

### 2.2. Heating and Cooling Procedures

**Figure 2:**
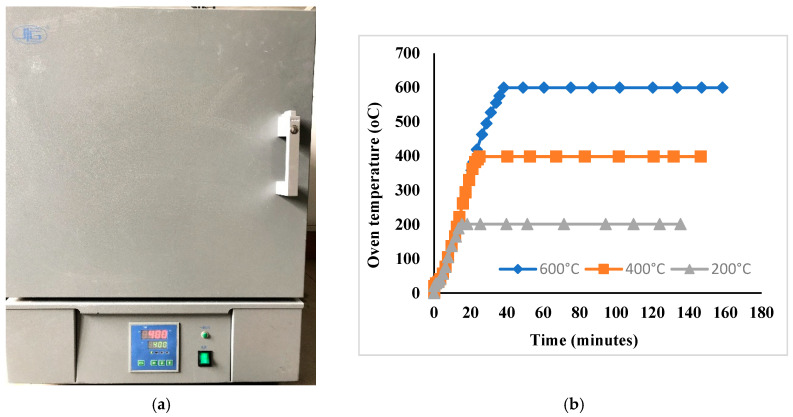
Electric furnace and time-temperature schedule: (**a**) Electric furnace; and (**b**) Time-oven temperature schedule.

To evaluate the effect of elevated temperatures on concrete surfaces, concrete specimens consisting of 150 × 150 × 200 mm prisms and 100 × 100 × 100 mm control cubes were heated in an electric furnace with a maximum temperature of 1000 °C. The heating protocol was designed to simulate fire exposure conditions, with a controlled heating rate of 10–15 °C/min until the target temperature was reached, as illustrated in Figure 2. The furnace temperature was controlled using the built-in furnace system during heating. Due to the closed furnace environment, direct measurement of specimen temperature during heating was not performed. An infrared thermometer (IR thermometer, Model DMQ304, DELIXI Electric Co., Ltd., Zhejiang, China) was used to measure the specimen surface temperature before and after the heating process to verify consistency with the furnace temperature. Although internal thermocouples were not used, the relatively small specimen size, together with the controlled heating rate and closed furnace conditions, was considered sufficient to ensure a reasonably uniform temperature distribution within the specimens.

The specimens were divided into three groups corresponding to target temperatures of 200 °C, 400 °C, and 600 °C. These temperature levels were selected based on previous studies indicating that concrete exposed to temperatures above 600 °C undergoes significant degradation, including a strength loss exceeding 40%, leading to brittle and irreversible damage [69]. To investigate the effect of exposure duration, each group was further subdivided into two subgroups subjected to heating durations of 1 h and 2 h, as summarized in Table 1. The furnace was heated at a controlled rate of approximately 10–15 °C/min until the target temperature was reached. Accordingly, the time required to reach the target temperature varied depending on the selected temperature level. Once the target temperature was reached, the specimens were maintained at that temperature for 1 or 2 h, depending on the experimental program.

After heating, the specimens were subjected to one of two cooling methods prior to CFRP bonding: Air cooling or water quenching [42,62]. The cooling rate was recorded during this stage. For air-cooled specimens, the average cooling rate from the target temperature to 100 °C was approximately 5–10 °C/min. In contrast, water quenching resulted in a rapid initial cooling rate exceeding 100 °C/min. For air cooling, the specimens were carefully removed from the furnace and allowed to cool naturally under ambient laboratory conditions (approximately 20–25 °C). For water quenching, the specimens were immediately submerged in a metallic tank (400 × 400 × 400 mm) filled with water at room temperature (20–25 °C). The water level was maintained at approximately 300 mm, corresponding to a volume of about 48 L, to ensure rapid heat dissipation. After 20–30 min, the specimens were removed from the water and placed in the laboratory to continue cooling at ambient temperature.

Following cooling, visual observations of the specimens were conducted. Specimens exposed to temperatures of 400 °C and above exhibited pronounced surface cracking and discoloration, while water-quenched specimens showed a denser network of fine cracks compared to air-cooled specimens. All specimens were then conditioned for one week before bond–slip testing.

**Figure 3:**
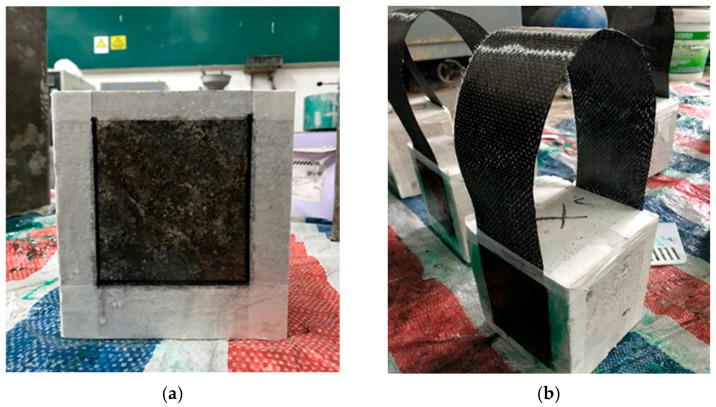
Preparing the concrete surface and bonding the CFRP sheets to the concrete surface: (**a**) Preparing the concrete surface; and (**b**) Bonding the CFRP sheets to the concrete surface.

The CFRP system was applied after the heating and cooling processes; therefore, the epoxy adhesive was not exposed to elevated temperatures during the fire simulation. Prior to bonding, the concrete surface was prepared by removing loose and deteriorated material using an abrasive brush and compressed air. Contaminants such as grease, dust, and surface residues were completely removed to ensure a clean bonding surface, as shown in Figure 3. The surface was then smoothed using fine-grit sandpaper, and the bonding area was marked.

Surface roughness was controlled through the standardized surface preparation procedure described above rather than by direct roughness measurement. This approach ensured consistent surface conditions across all specimens. The adhesive thickness was controlled during application by applying a uniform epoxy layer and pressing the CFRP sheets using a plastic roller along the fiber direction to promote even resin distribution. Although the adhesive thickness was not measured using a dedicated gauge, the same application procedure was followed for all specimens to maintain consistency and repeatability.

The CFRP sheets, with a width of 100 mm and 25 mm unbonded zones at each end, were prepared as illustrated in Figure 3a. The epoxy resin was mixed for 3 min and ap-plied uniformly to the prepared concrete surface. The CFRP sheets were then placed and pressed using a plastic roller along the fiber direction to ensure proper impregnation and bonding. The 150 × 150 × 200 mm prisms were oriented with the 200 mm dimension aligned with the loading direction.

### 2.3. Test Setup and Instrumentation

**Figure 4:**
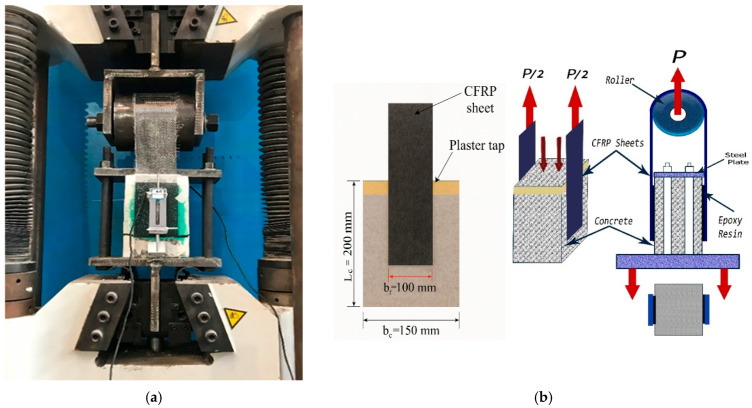
Test setup and configuration of the double-lap direct shear test: (**a**) Photograph of the test setup; and (**b**) Schematic illustration of the specimen configuration and dimensions.

A double-lap direct shear test was performed using a standard testing machine in accordance with HB305-2008 [70,71,72,73,74]. The test applied shear forces on both sides of the specimens. The testing machine exerted an upward force on a U-shaped steel plate connected to an aluminum cylinder, which pulled the central section of the CFRP sheet upward. This action generated shear forces at the bonded interfaces between the CFRP sheets and the concrete surfaces on both sides. The concrete blocks were securely fixed to the machine base using a steel plate equipped with anchors. The tests were conducted at a loading rate of 0.5 mm/min under displacement control. Two linear variable differential transducers (LVDTs) were installed on both sides of the blocks to measure the load–slip relationship between the CFRP sheets and the concrete blocks. Each LVDT was positioned between two specific points: One at the top, the other at the bottom of the concrete block. Load-slip data were recorded using the pull-out testing system and the LVDTs. These measurements were then synchronized and analyzed to ensure accuracy and consistency. The pull-out test instrumentation and test configuration are shown in Figure 4.

## 3. Results and Discussion

### 3.1. Compressive Strength at Elevated Temperatures

#### 3.1.1. Visual Observation of Cracking Extent

Table 4 shows the cracking patterns and their corresponding extents observed in concrete specimens after exposure to 200 °C, 400 °C, and 600 °C, followed by cooling. The extent of damage to the concrete was estimated based on visual inspection. Assessing fire-damaged concrete typically begins with evaluating observable changes, such as variations in color, surface cracking, or spalling. In this investigation, observations during heating and cooling indicated noticeable color changes in the concrete, which were influenced by the exposure temperature. These color changes may be associated with alterations in texture and composition, as well as possible changes in the internal structure of the material during heating and cooling.

The observed changes were further likely related to the expansion and evaporation of absorbed water during heating. During cooling, it is suggested that ionized CaO produced from the decomposition of Ca (OH)_2_ may react with water and partially reform Ca (OH)_2_. As a result of this reaction, the concrete may experience slight expansion, which could influence internal cracks and voids and potentially affect its durability and sealing performance [75,76,77].

In the water-cooled specimens, thermal shock occurred due to rapid cooling and water interaction. However, damage continued to develop during the cooling process, reducing the residual strength of the concrete and leading to progressive crack growth over time. Specimens exposed to 200 °C showed the fewest surface cracks, but the number and width of cracks increased significantly with higher exposure temperatures. At 400–600 °C, the concrete prisms exhibited the most severe cracking. In addition to cracking, color changes provided important visual indicators of thermal damage, reflecting possible alterations in material composition and surface texture due to elevated temperatures [77]. Table 4 further presents how temperature, fire-exposure duration, and cooling method influence the color changes in concrete. For example, at 200 °C, the concrete displayed a subtle shift to light gray. At 400 °C, the color became noticeably darker, transitioning to dark gray at 600 °C, accompanied by small black spots visible on the surface of the specimens.

Furthermore, the results presented in Table 4 indicate that differences in concrete surface color and cracking may be influenced by the combined effects of thermal exposure and cooling rate. According to previous studies, higher furnace temperatures (400–600 °C) are associated with the dehydration of the cement paste and possible decomposition of Portlandite (Ca (OH)_2_) into CaO, along with degradation of C–S–H gel. These changes may contribute to variations in surface appearance, including whitish or reddish discoloration. Prolonged exposure (2 h) may intensify these effects. From a physical perspective, the cooling method appears to play an important role. Rapid water quenching may induce thermal shock, generating steep temperature gradients and tensile stresses, which can lead to the development of additional microcracks in the thermally affected matrix and at the aggregate–paste interface. In contrast, gradual air cooling allows more uniform stress redistribution, resulting in fewer and wider cracks. The combination of high temperature and rapid cooling is therefore associated with more severe visible damage, including increased cracking and more pronounced color changes.

#### 3.1.2. Evaluation of Residual Concrete Strength

**Figure 5:**
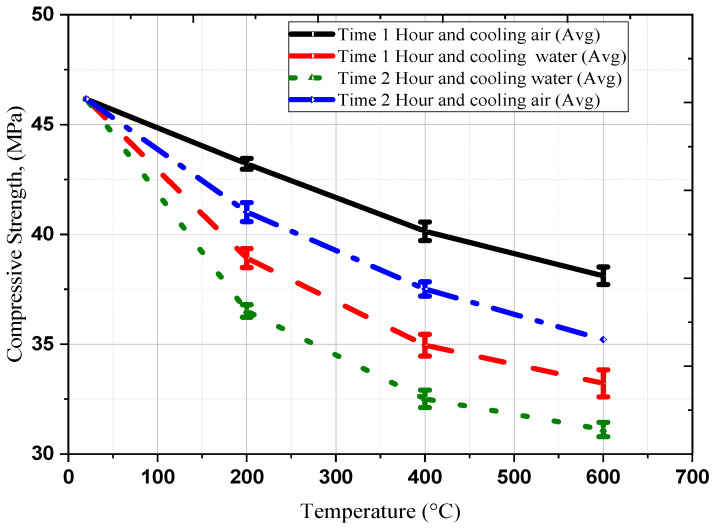
Residual Compressive Strength with Variability (*n* = 2–3 per-group).

Figure 5 depicts the residual compressive strengths of concrete after exposure to various temperatures, durations, and cooling methods (air and water). The results represent the average of three replicate experiments. In addition, trends in residual compressive and splitting strengths relative to oven temperature demonstrated the effect of high-temperature exposure on concrete. The curves for different concrete groups showed nearly identical behavior, with a slight decrease in strength at 200 °C followed by a substantial decline at higher temperatures. Elevated temperatures exceeding 400 °C likely contributed to strength deterioration, attributed to thermally induced map-type cracks or the decomposition of cement binding materials. Exposure and cooling methods significantly affected the reduction in residual compressive and splitting strengths, ranging from 94% at 200 °C to 53% at 600 °C, as summarized in Table 5.

Table 2 shows the test results of concrete compressive strengths, including average values and SD. The SD values are also shown in Figure 5 as error bars, representing the variability in compressive strength measurements across three replicate specimens for each experimental temperature and cooling condition. SD provides a measure of dispersion, indicating how consistently the material behaves under identical experimental conditions.

At room temperature (20 °C), all specimens showed almost closely compressive strength values with an average of 46.16 MPa, demonstrating perfect repeatability under baseline conditions and confirming precise experimental control. At elevated temperatures (200–600 °C), air-cooled specimens showed higher consistency compared to water-cooled specimens. For 1 h air cooling, the SD values increased from 0.245 MPa at 200 °C to 0.424 MPa at 400 °C, indicating greater variability in residual strength of concrete as the temperature rose. Conversely, water cooling introduced higher uncertainty at elevated temperatures. For instance, at 600 °C, the SD for 1 h treatment increased to 0.616 MPa, compared to 0.400 MPa for air cooling at the same temperature. This heightened variability may be attributed to nonuniform thermal gradients or rapid water cooling, which can cause localized stress gradients or moisture absorption, leading to greater variability. Specifically, for 1 h water cooling, the SD increased from 0.432 MPa at 200 °C to 0.616 MPa at 600 °C. The greater variability is likely attributable to higher thermal stresses causing microstructural inhomogeneity (e.g., grain boundary weakening).

Missing data points, such as SD values for *n* = 2, may underestimate the true variability due to a limited sample size. Therefore, future studies should include at least five replicates (*n* ≥ 5) at high temperatures to improve data accuracy.

### 3.2. Failure of the CFRP–Concrete Bond

**Figure 6:**
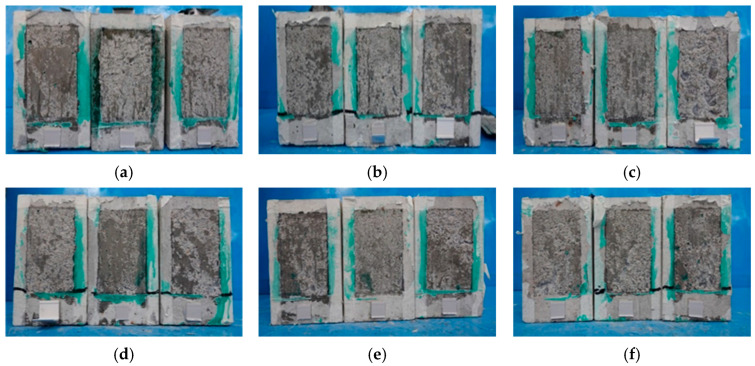

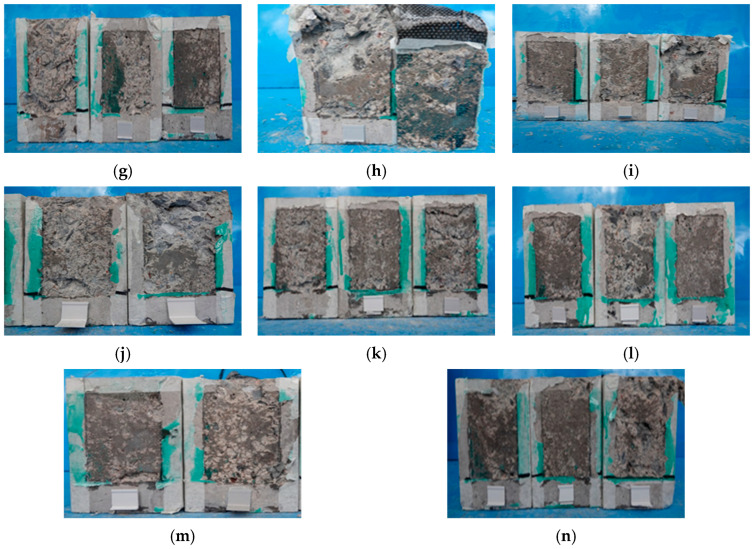
Failure between CFRP sheets and concrete substrate: (**a**) Control Specimens; (**b**) 200 °C-One Hour-Air; (**c**) 200 °C-One Hour-Water; (**d**) 200 °C-Two Hour-Air; (**e**) 200 °C-Two Hour-Water; (**f**) 400 °C-One Hour-Air; (**g**) 600 °C-One Hour-Air; (**h**,**i**) 600 °C-One Hour-Water; (**j**) 400 °C-One Hour-Water; (**k**) 400 °C-Two Hour-Air; (**l**) 400 °C-Two Hour-Water; (**m**) 600 °C-Two Hour-Air; and (**n**) 600 °C-Two Hour-Water.

The failure mechanism observed across all tested specimens followed a consistent pattern. As the applied force increased, the CFRP sheets became increasingly taut. When the load reached approximately 80% of its peak value, an audible popping sound signaled the initiation of localized debonding at certain points within the bond interface. As the force approached its maximum threshold, the CFRP sheets experienced longitudinal tearing, accompanied by a shattering sound at the loaded end. The final stage of failure was characterized by the complete detachment of the CFRP sheets from the concrete surface, as depicted in Figure 6. The debonding failure between the CFRP sheets and the concrete was sudden and brittle, releasing a significant amount of energy, which led to the longitudinal splitting of the CFRP sheets. Visual inspection of the failed specimens revealed that only a minimal amount of concrete remained attached to the detached CFRP sheets, indicating an adhesive failure mode rather than a cohesive failure within the concrete substrate.

The failure behavior varied depending on the temperature exposure and cooling method. For specimens previously exposed to temperatures of 200–400 °C, the bond failure primarily occurred via adhesion, where the CFRP sheets detached from the concrete surface, leaving some residue of adhesive material. At higher temperatures (600 °C), the specimens exhibited severe damage, with extensive concrete spalling and cracking, which weakened the bond and facilitated premature CFRP detachment. In water-cooled samples, the rapid thermal shock further accelerated delamination and promoted faster degradation of the bond interface. Failure occurred predominantly on one side of the specimens, suggesting an asymmetric stress distribution or localized weak points in the bond. Once failure occurred—whether due to fiber rupture or bond failure—the testing equipment immediately ceased data acquisition, marking the end of the load–slip measurement process, and the data collection was manually stopped after the test was stopped.

### 3.3. Load–Slip Relationships of CFRP–Concrete Bond

**Figure 7:**
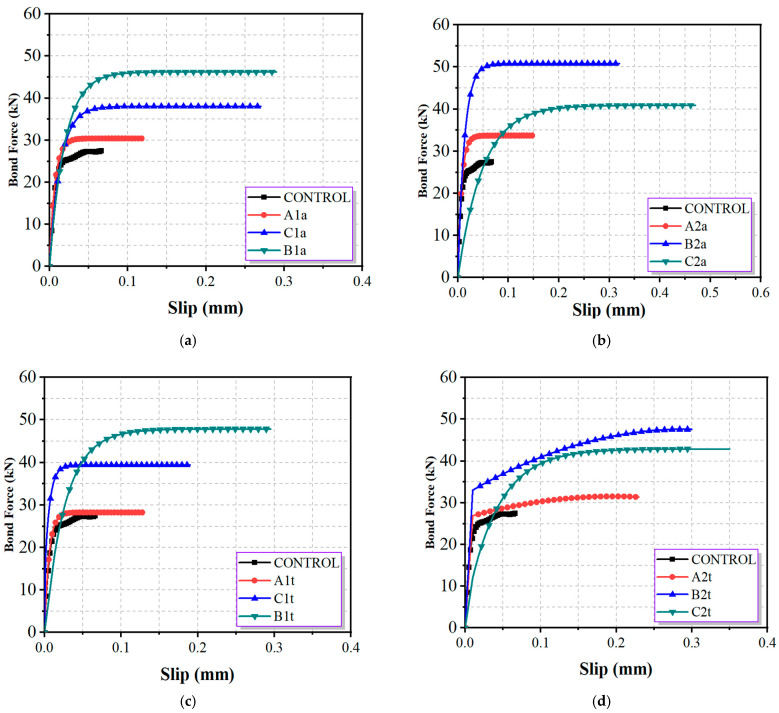
Bond force–slip relationships of CFRP–concrete bonded specimens after exposure to elevated temperatures followed by cooling methods and durations: (**a**) 1 h air cooling, (**b**) 2 h air cooling, (**c**) 1 h water cooling, and (**d**) 2 h water cooling.

Figure 7 presents four subfigures that compare specimens based on the duration of fire exposure and cooling methods. The load–slip curves for the double-shear specimens in this experiment could be identified in two stages. In Stage 1, the relationship between load and slip is linear, as the CFRP sheets and the concrete bonding interface remained intact, causing slip to increase proportionally with the applied load. In Stage 2, the load–slip curves increased nonlinearly, indicating the onset of degradation in the bond interface.

As shown in Table 6, the bond force increased with temperature up to 400 °C. However, beyond this temperature threshold, the increase in bond force became less pronounced, as observed at 600 °C. Nonetheless, the slip generally increased with the temperature. Furthermore, as shown in Table 6, increasing bonding force ratios for various specimens compared to control samples highlights the significant impact of treatment temperature, the cooling method, and duration on bonding force enhancement. For example, the S200 series demonstrated modest improvements, peaking at a 21.4% increase with a 2 h air cooling, while water cooling yielded less than a 10% gain. In contrast, the S400 series showed the most substantial enhancements, with bonding force increases of 46.0% and 79.2% for 1 h and 2 h air cooling, respectively. Water cooling also proved effective, resulting in increases of 42.0% and 66.3%. Interestingly, the S600 series displayed a different trend; longer air-cooling durations led to a slight decrease in bonding force from 41.8% to 37.9%. However, the water-cooled specimens responded positively to extended cooling time, increasing from 34.2% to 56.8%.

The obtained load–slip responses exhibit a characteristic behavior consisting of an initial linear stage followed by a nonlinear softening stage, which is consistent with observations reported in previous studies on CFRP–concrete interfaces after thermal exposure. For instance, previous studies [63] reported that elevated temperatures lead to a reduction in bond strength and an increase in slip at failure, reflecting degradation in interfacial stiffness and fracturing energy. Similarly, other researchers [78] observed that increasing temperature results in a transition in failure modes and a reduction in peak load due to interfacial damage mechanisms. In addition, studies on post-fire concrete–resin interfaces [79] demonstrated that the bond–separation behavior is governed by progressive cracking and energy dissipation processes influenced by the thermal history of the substrate. Comparable trends were also reported in the literature [80], where bond capacity degradation in heat-damaged concrete was closely associated with microstructural deterioration and reduced substrate integrity.

Overall, although the present study does not explicitly employ a fracture mechanics-based formulation, the observed load–slip behavior and failure mechanisms are consistent with the fundamental fracture-based interpretations reported in the literature. This confirms that the proposed interpretation of the bond–slip response remains physically meaningful and aligned with established interfacial behavior of CFRP-strengthened concrete systems under thermal damage conditions.

### 3.4. Investigating the Impact of Different Parameters on Bond Behavior

#### 3.4.1. Influence of Different Oven Temperatures on Bond Strength

**Figure 8:**
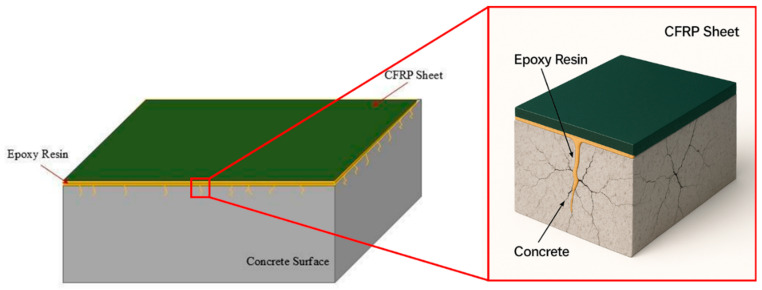
Representation of epoxy penetration into the concrete surface.

**Figure 9:**
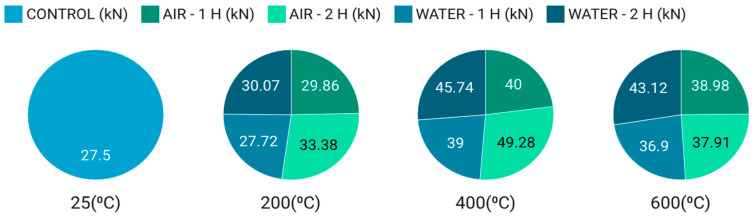
Diagram of the maximum tensile strength with oven temperature, duration of exposure to heating, and specimen cooling method.

**Figure 10:**
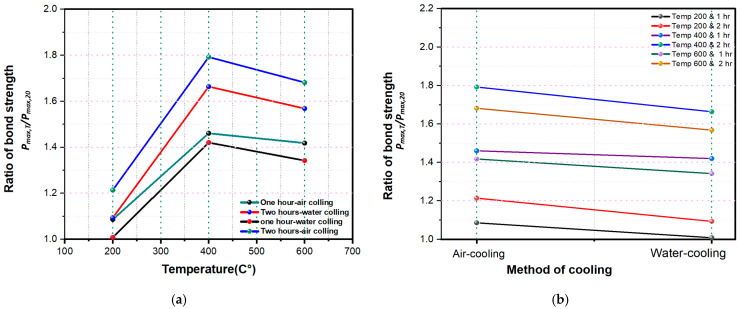
Effect of different oven temperatures and exposure time on bond strength: (**a**) Effect of different oven temperatures, (**b**) Effect of exposure time to fire.

The bond strength between the CFRP sheets and concrete was directly affected by increasing temperature. At 200 °C, a slight rise in bond strength was observed relative to room temperature. As the temperature increased further (400–600 °C), the bond strength showed additional improvement. This enhancement may be attributed to the widening of cracks on the concrete surface at elevated temperatures, which allows the resin to penetrate more deeply into the concrete (Figure 8), thereby improving the bond between the concrete and CFRP sheets (Figure 9). This result is consistent with [46]. Figure 10a presents the relationship between temperature and the bond strength ratio (***P***max, T/***P***max,20), where $P_{max,T}$ is the bond strength at temperature T (°C) and *P*max,20 is the bond strength at 20 °C (room temperature). The bond strength ratio for fire-damaged concrete–CFRP interfaces increased with temperature up to 400 °C, after which it began to decrease. In detail, increasing the temperature enhanced the bond strength ratio: under one hour of air cooling, the ratio was 1.09% at 200 °C, and it increased to approximately 34.50% and 30.56% at 400 °C and 600 °C, respectively. Moreover, the rise in oven temperature had a more pronounced effect on bond strength for specimens exposed to fire for 2 h and air-cooled than for specimens exposed for 1 h and water-cooled. Accordingly, temperature exerts a significant influence on the bond-strength behavior of fire-damaged concrete.

#### 3.4.2. Influence of Exposure Duration on Bond Strength

The experimental results show that the bond strength between the concrete and CFRP sheets was affected by the duration of exposure to high oven temperatures. Specifically, specimens exposed to fire for 2 h exhibited greater bond strength than those exposed for 1 h. This suggests that the bond strength of specimens heated for 2 h increased with temperature. Table 7 indicates that the slip observed in specimens exposed to fire for 2 h was more significant than those exposed for 1 h. Figure 10b shows the effect of fire exposure time and cooling method on the bond strength ratio (*P*_max_/*P*_max0_) at different elevated temperatures, where “1” represents air cooling, and “2” represents water cooling. In more detail, increasing the duration of fire exposure improved the bond strength ratio. Specifically, at 200 °C with air cooling, the bond strength ratio was 1.09% after 1 h of exposure and increased to 1.21% after 2 h. In general, specimens exposed to longer heating durations (2 h) exhibited lower bond strength ratios than those exposed for only 1 h, indicating that prolonged exposure to elevated temperatures further degrades bond performance. Additionally, the water-cooling method (“2”) consistently led to a lower bond strength ratio than air cooling (“1”) across all temperature levels and exposure times. This trend can be attributed to the thermal shock introduced by rapid cooling, which intensifies microcracking and weakens the interfacial bond. The reduction in bond strength is more significant at higher temperatures (e.g., 600 °C), emphasizing the detrimental impact of both high thermal exposure and aggressive cooling. These findings highlight the importance of considering both exposure duration and cooling strategy in post-fire assessment and in the design of structural retrofitting measures.

#### 3.4.3. Influence of Cooling Method on Bond Strength

As mentioned earlier, two cooling methods were adopted in this research, namely air cooling and water cooling. The experimental findings demonstrated that air cooling generally preserved bond strength more effectively than water cooling. This can be attributed to the slower cooling rate of air, which allowed specimens to return gradually to ambient temperature and thereby reduced thermal shock. In contrast, the rapid quenching associated with water cooling induced steep temperature gradients, internal microcracking, and increased porosity within the concrete surface, all of which adversely affected the bond at the CFRP–concrete interface.

As presented in Table 7, specimens subjected to air cooling consistently exhibited higher bond strength values across all temperature levels compared to those cooled in water. At 200 °C and 400 °C, air-cooled specimens retained superior bond strength due to the gradual dissipation of heat, which mitigated excessive thermal stress and crack propagation. By contrast, water-cooled specimens experienced a greater reduction in bond strength, highlighting the detrimental effects of rapid cooling on the CFRP–concrete interface. With further elaboration, when the temperature was fixed at °C and the duration at one hour, the bond strength ratio decreased by approximately 7% as the cooling method shifted from air cooling to water cooling, as shown in Figure 10b. In the same manner, the bonding force declined from 29.86 kN to 27.72 kN under the same cooling change, as presented in Table 7.

At 600 °C, an unexpected trend was observed: water-cooled specimens displayed partial bond strength recovery, in some cases surpassing air-cooled specimens. This phenomenon may be attributed to enhanced surface roughness and microcracking-induced mechanical interlocking, which improved adhesion in severely damaged specimens. Although water cooling generally weakened the bond due to rapid contraction and cracking, at extreme temperatures, the increased roughness of the concrete may contribute to localized mechanical interlock, partially compensating for the thermal degradation effects. These results align with findings from previous studies [81], emphasizing that post-fire cooling methods not only influence residual bond strength but also affect failure mechanisms and interfacial adhesion properties. The observed variations underscore the importance of considering cooling strategies when assessing the structural performance of CFRP-strengthened, fire-damaged concrete.

## 4. Analytical Investigations

### 4.1. Development of Equations for Bond Strength at Elevated Temperatures

The equations proposed in the literature for predicting bond strength between fire-damaged concrete and CFRP sheets considered only limited parameters. Therefore, this study proposes a new equation that incorporates the influence of additional factors, particularly the cooling method. Based on an analysis of the effect of these parameters on the bond behavior, an equation in the form of $P_{perd,\;T}=∆\alpha_{1}\alpha_{2}\alpha_{3}\beta_{p}\beta_{L}\sqrt{f_{cT}^{\prime }}b_{p}L_{eT}$ was proposed to predict the bond strength at elevated temperatures. Parameters $\alpha_{1}$, $\alpha_{2}$ and α_3_ consider the effects of temperature, duration of exposure to fire, and method of cooling, respectively, and variables $\beta_{p}$, $\beta_{L}$ and $L_{eT}$ represent the width and length of the CFRP sheets and the effective bond length, respectively. The parameter ∆ is calculated using a regression analysis of the predicted and experimental bond strength between the fire-damaged concrete and CFRP sheets. The final form of the proposed equation is presented in Equation (1).

(1) $$P_{Pro,T}=0.086\alpha_{1}\alpha_{2}\alpha_{3}\beta_{p}\beta_{L}\sqrt{f_{cT}^{\prime }}b_{p}L_{eT},$$

(2) $$\alpha_{1}=\left\{\begin{array}{cc} 0.0024T+0.62 & if\;20\;{}^{\circ}C<T\leq 400\;{}^{\circ}C\\ -0.0004T+1.75 & if\;400\;{}^{\circ}C\leq T\leq 600\;{}^{\circ}C \end{array}\right\},$$

(3) $$\alpha_{2}=0.213t+1.076,$$

(4) $$\alpha_{3}=\left[\begin{array}{cc} 1.44 & if\;use\;cooling\;with\;air\\ 1.35 & if\;use\;cooling\;with\;water \end{array}\right],$$

where T denotes the temperature; t is the duration of exposure to fire; $f_{cT}^{\prime }$ is the compression strength of concrete at any temperature between 20 °C and 600 °C [82] and $\beta_{p}$ is the influence coefficient of the CFRP pasting width, where $b_{p}$ is the width of the CFRP paste, and $b_{c}$ is the width of the concrete specimen.

(5) $$\beta_{p}=\sqrt{\frac{2-\frac{b_{p}}{b_{c}}}{1+\frac{b_{p}}{b_{c}}}}$$

Equation (5) is from the study of Chen and Teng [83]. $\beta_{L}$ is the influence coefficient of the CFRP pasted length, which can be expressed as follows:

(6) $$\beta_{L}=\left[\begin{array}{c} 1\;\;\;\;\;\;\;\;\;\;\;\;\;\;\;\;\;\;\;\;if\;\;(L\geq L_{e})\\ \sin\frac{\pi L}{2L_{e}}\;\;\;\;\;\;\;\;\;\;\;\;if\;(L<L_{e}) \end{array}\right],$$

where *L* is the bonding length of the CFRP, and $L_{e}$ is the effective bonding length between the CFRP and concrete. $L_{e}$, also referred to as the active bond length, is the portion of the bond where most of the bond stress is maintained [84].

(7) $$L_{e}=\frac{23300}{{\left(n_{f}t_{f}E_{f}\right)}^{0.58}},$$

where $n_{f}$ is the number of layers, $t_{f}$ represents thickness, and $E_{f}$ denotes the Young’s modulus of CFRP.

### 4.2. Validation of Bond Strength Using the Proposed Equation

**Figure 11:**
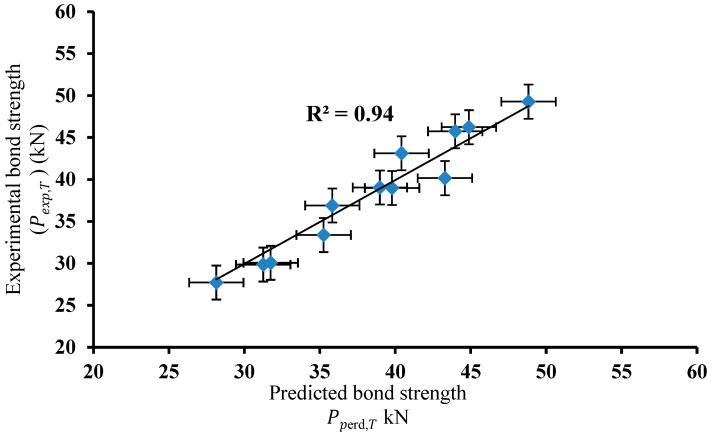
Relationship between experimental and predicted bond strength.

The ratio between the predicted and the experimental bond strength was calculated for all specimens, as shown in Table 8. To evaluate the accuracy of the proposed equation, the relationship between the experimental and predicted bond strength for the fire-damaged concrete and CFRP sheets was plotted, as shown in Figure 11. The proposed equation demonstrated high accuracy (*R*^2^ = 0.94) in predicting bond strength at different temperatures (Figure 11) and yielded predictions with minimal error (Table 8). The proposed statistical equation provided the most accurate and reliable results, with an average ratio of analysis to test results (*P_perd,T_/P_exp,T_*) and COV of 1.015 and 0.044, respectively, across different concrete types and temperatures. By contrast, the model equation by [85] showed significantly less accurate predictions, with an average ratio (*P**_u_**_(Wu)_/P_exp,T_*) and COV of 0.456 and 0.34, respectively, for the same data. In addition, the *t*-test values were calculated to compare the data from the proposed model in this study with the test results, as well as the data from the model by Wu et al. [85] with the test results, as shown in Table 9 and Table 10. The *t*-test value and *p*-value for the proposed model are smaller than those for [85], indicating that the means of the two groups are closer, resulting in more accurate predictions. Generally, values closer to zero suggest greater similarity between groups and more reliable predictions, whereas values further from zero suggest a larger difference between the means. The *t*-test statistics can take on any positive or negative value. Table 8 shows the *t*-test analysis assuming unequal variances for the test data and this study’s model, whereas Table 10 provides a similar analysis for the Wu et al. [85] model referenced against the test data. It should be noted that the validation of the proposed model is primarily based on the comprehensive experimental dataset developed in this study. This approach was adopted due to the limited availability of independent datasets in the literature. In most cases, peak temperature, exposure duration, and cooling method are not considered simultaneously and in a systematic manner. While the model captures the observed experimental trends with good agreement, further validation using independent datasets is recommended in future work to enhance its general applicability across different material properties and structural configurations.

## 5. Conclusions

This study investigated the bond behavior between CFRP sheets and fire-damaged concrete, with particular emphasis on the combined effects of temperature level, fire exposure duration, and post-fire cooling method. A total of 39 specimens were tested using double-lap joint configurations, including unheated control specimens and specimens exposed to temperatures of 200 °C, 400 °C, and 600 °C for durations of one and two hours, followed by either air cooling or water cooling before CFRP application. Based on the experimental results and analytical evaluation, the following conclusions can be drawn:

1. The severity of material damage exhibited a positive correlation with the heating temperature, reaching its maximum at 600 °C, where specimens experienced the most extensive deterioration. The compressive strength progressively declined with increasing temperature and prolonged exposure, indicating cumulative thermal degradation. This reduction was particularly pronounced in specimens subjected to two hours of heating, suggesting that extended thermal exposure accelerates processes such as crack propagation, dehydration, and loss of cohesive bonding. Furthermore, the decline in compressive strength was exacerbated under water-cooling conditions. The abrupt quenching introduced by water cooling induced thermal shock, which intensified cracking and spalling, thereby compounding the deterioration compared with air-cooled counterparts.
2. Water-cooled specimens exhibited a greater reduction in residual compressive strength than air-cooled specimens, owing to more extensive cracking under water-cooling, which highlights the negative effect of thermal shock on concrete integrity. The longer the exposure, the more significant the reduction in compressive strength, emphasizing the degradation caused by prolonged heating as cracks expanded rapidly with increasing heating duration. Standard deviation (SD) error bars robustly quantified experimental variability and revealed critical trends.
3. The bond strength between the CFRP sheets and concrete initially increased with temperature. This may be attributed to crack widening, which facilitated greater epoxy penetration into the concrete substrate, enhancing adhesion. This effect was observed up to 400 °C, beyond which bond strength declined due to severe material degradation.
4. Bond strength is strongly affected by temperature. An increase in temperature led to an improvement in bond strength because wider cracks allowed the resin to penetrate more deeply into the concrete. Similarly, exposure duration shows an inverse correlation with bond strength, longer fire exposure results in remarkable degradation of bond strength. The cooling method also significantly influenced bond strength, with air-cooled specimens exhibiting better bond retention than water-cooled specimens. Thermal shock from water immersion accelerated concrete weakening and thus further degraded the bond strength.
5. The proposed analytical model demonstrated high accuracy in predicting bond strength across various fire-exposure conditions, achieving an R^2^ of 0.94, an analysis-to-test ratio (*P_p_*_erd*,T*_/*P_exp,T_*) of 1.015, and a COV of 0.044. Statistical analysis (*t*-test and *p*-values) confirmed that the proposed model provides a more reliable prediction of bond strength, making it a valuable tool for evaluating CFRP-strengthened, fire-damaged concrete systems.

The findings of this study provide important insights into the effectiveness of CFRP in strengthening fire-damaged concrete structures, offering direct implications for post-fire rehabilitation and structural performance assessment. Specifically, the results emphasize the critical influence of temperature exposure and cooling methods on bond behavior and residual strength, underscoring the role of CFRP as a reliable strengthening technique for fire-affected concrete. Furthermore, the proposed predictive equation offers a scientifically validated tool for estimating bond strength variations in CFRP-strengthened, fire-damaged concrete, enabling engineers to make more accurate evaluations and optimize repair strategies. In addition, this research advances the development of improved rehabilitation practices that promote the safe and efficient reuse of fire-damaged concrete structures. Future work could extend these findings by examining the effectiveness of various fire-protective coatings, exploring the influence of different aggregate types, and assessing the size effect of specimens on the bond durability of CFRP-strengthened, fire-damaged concrete. Moreover, the development of an equation that incorporates all these parameters collectively would enhance predictive accuracy. Finally, long-term studies focused on the mechanical performance and durability of CFRP-strengthened concrete after fire exposure and subsequent rehabilitation would provide further insight into its sustained structural performance. This study advances the understanding of CFRP-concrete bond behavior after fire by systematically quantifying the critical, yet previously underexplored, role of the cooling method. While it is known that bond strength is influenced by temperature, our findings demonstrate that the post-heating cooling regime is not merely a procedural detail but a dominant factor governing residual bond performance. The consistent reduction in bond strength observed in water-cooled specimens compared to their air-cooled counterparts, attributable to thermal shock-induced microcracking, provides a crucial insight for post-fire assessment protocols. Moreover, the unexpected partial recovery of bond strength in water-cooled specimens at 600 °C introduces a new nuance to the failure mechanisms, suggesting that extreme damage can, in some cases, enhance mechanical interlock. By integrating temperature, exposure duration, and cooling method into a single, highly accurate predictive model (R^2^ = 0.94), this work moves beyond qualitative descriptions to offer a quantitative framework. This represents a significant step forward, enabling engineers to make more informed and reliable decisions in the rehabilitation of fire-damaged structures, ultimately bridging the gap between laboratory research and practical post-fire engineering strategies.

## Figures and Tables

**Table 1:** Details of the specimen experimental program.

Specimens Group	Notation	Number of Specimens	Heating Oven Temperature Degree (°C)	Duration of Exposure to Fire (h)	Cooling Method
Control specimens	Control-1	3	20	None	-
A	A1a	3	200	1	Air
	A1t	3		1	Water
	A2a	3		2	Air
	A2t	3		2	Water
B	B1a	3	400	1	Air
	B1t	3		1	Water
	B2a	3		2	Air
	B2t	3		2	Water
C	C1a	3	600	1	Air
	C1t	3		1	Water
	C2a	3		2	Air
	C2t	3		2	Water

**Table 2:** Test results of concrete compressive strengths, average, and SD.

Temp. (°C)	Fire Exposure Time (h)—Cooling Method	Specimen 1	Specimen 2	Specimen 3	Average (MPa)	Standard Dev.
20	1 h-Air	46.16	46.16	46.16	46.16	0.000
200	1 h-Air	43.51	42.91	43.21	43.21	0.24
400	1 h-Air	40.74	39.84	39.84	40.14	0.42
600	1 h-Air	37.72	—	38.51	38.11	0.40
20	1 h-Water	46.16	46.16	46.16	46.16	0.00
200	1 h-Water	39.32	38.32	39.12	38.92	0.43
400	1 h-Water	34.26	35.36	35.26	34.96	0.50
600	1 h-Water	32.52	33.12	34.02	33.22	0.62
20	2 h-Air	46.16	46.16	46.16	46.16	0.00
200	2 h-Air	40.62	41.62	40.82	41.02	0.43
400	2 h-Air	37.91	37.11	37.51	37.51	0.33
600	2 h-Air	35.21	—	35.21	35.21	0.00
20	2 h-Water	46.16	46.16	46.16	46.16	0.00
200	2 h-Water	36.91	36.31	36.31	36.51	0.28
400	2 h-Water	32.91	32.11	—	32.51	0.40
600	2 h-Water	31.52	30.72	31.12	31.12	0.33

Note: “—” indicates missing or unavailable data.

**Table 3:** Tensile properties of CFRP sheets.

Property	Value	Unit	Standard
Tensile strength	3500	MPa	ASTM D3039 [66]
Elastic modulus	250	GPa	ASTM D3039 [66]
Ultimate strain	1.4	%	ASTM D3039 [66]
Fiber thickness (nominal)	0.167	mm	—
Laminate thickness (measured)	~1.0	mm	—

**Table 4:** Concrete surface after fire.

Oven Temperature (°C)	1 h-Air	1 h-Water	2 h-Air	2 h-Water
20°C	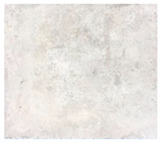			
200°C	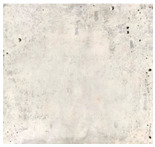	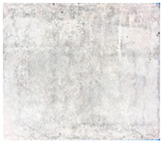	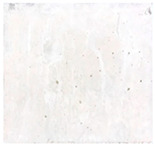	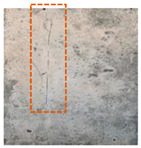
400°C	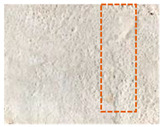	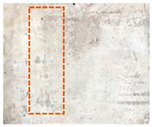	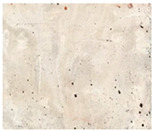	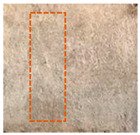
600°C	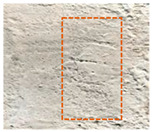	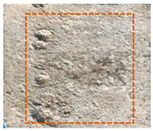	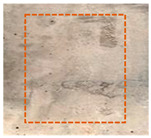	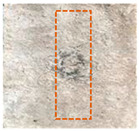

**Table 5:** Residual Compressive Strength of Concrete under Thermal and Cooling Conditions.

Specimen No.	Temperature (°C)	Compressive Strength (MPa)			
		Air-1 h	Water-1 h	Air-2 h	Water-2 h
S20	20	46.16	46.16	46.16	46.16
S200	200	43.21	38.92	41.02	36.51
S400	400	40.14	34.96	37.51	32.51
S600	600	38.11	33.22	35.21	31.12

**Table 6:** Increasing ratio in bonding force compared to control specimens.

Specimen No	Temperature (°C)	1 h-Air Cooling	2 h-Air Cooling	1 h-Water Cooling	2 h-Water Cooling
S200	200	8.6%	21.4%	0.8%	9.3%
S400	400	46.0%	79.2%	42.0%	66.3%
S600	600	41.8%	37.9%	34.2%	56.8%

**Table 7:** Maximum bond strength of CFRP–concrete specimens under thermal–cooling conditions.

Specimens Group	Notation	Heating Oven Temperature (°C)	Duration of Exposure to Fire (h)	Cooling Method	Average Maximum Pull-Out Test Force (kN)
Control specimen	Control	20	None	-	27.50
A	A1a	200	1	air	29.86
	A1t		1	water	27.72
	A2a		2	air	33.38
	A2t		2	water	30.07
B	B1a	400	1	air	40.16
	B1t		1	water	39.05
	B2a		2	air	49.28
	B2t		2	water	45.74
C	C1a	600	1	air	38.98
	C1t		1	water	36.90
	C2a		2	air	37.91
	C2t		2	water	43.12

**Table 8:** Validation and comparison of the proposed bond strength equation.

Ref./Author	Specimen Label (Notation)	Temperature (°C)	Duration of Exposure to Fire (h)	Cooling Method	*P_exp,T_* (kN)	This Study		Wu et al. [85]		
						*P_p_*_erd*,T*_ (kN)	*P_p_*_erd*,T*_/*P_exp,T_*	P_u(Wu)_ (kN)	P_u(Wu)_/P*_exp,T_*	P_u(this study_/P_u(Wu)_
This Study	Control-1	20	-	-	27.50					
	A1a	200	1	air	29.86	31.25	1.05	14.63	0.49	2.13
	A1t	200	1	water	27.72	28.14	1.01	14.48	0.52	1.94
	A2a	200	2	air	33.38	35.25	1.06	14.56	0.44	2.42
	A2t	200	2	water	30.07	31.74	1.05	14.39	0.48	2.21
	B1a	400	1	air	40.16	43.29	1.08	14.52	0.36	2.98
	B1t	400	1	water	39.05	38.98	0.99	14.33	0.37	2.72
	B2a	400	2	air	49.28	48.83	0.99	14.43	0.29	3.38
	B2t	400	2	water	45.74	43.97	0.96	14.22	0.31	3.09
	C1a	600	1	air	38.98	39.79	1.02	14.45	0.37	2.75
	C1t	600	1	water	36.90	35.83	0.97	14.25	0.39	2.51
	C2a	600	2	air	46.24	44.88	0.97	14.34	0.31	3.13
	C2t	600	2	water	43.12	40.41	0.94	14.16	0.33	2.85
Haddad et al. [44]	LWAC	20	2	air	20.55	20.93	1.02	14.18	0.69	1.48
	NWAC (1)	20	2	air	21.55	21.49	0.99	14.25	0.66	1.51
	NWAC (2)	20	2	air	16.50	18.19	1.10	13.78	0.83	1.32
Mean							1.015	14.33	0.46	2.43
Standard deviation							0.045	0.201	0.155	0.626
Coef. Of var. = stand. Dev./mean							0.044	0.014	0.340	0.258

**Table 9:** *t*-test: Two samples assuming unequal variances, test data, and this study of experimental to predicted values.

Statistics	Variable 1 (*P_exp,T_* (kN))	Variable 2 (*P_p_*_erd*,T*_ (kN))
Mean	34.608	34.865
Variance	100.729	88.659
Observations	15	15
Hypothesized Mean Difference	0	
df	28	
t Stat	−0.072	
*P* (T ≤ t) one-tail	0.471	
t Critical one-tail	1.701	
*P* (T ≤ t) two-tail	0.942	
t Critical two-tail	2.048	

**Table 10:** *t*-test: Two samples assuming unequal variances, test data, and this study of experimental to Wu values.

Statistics	Variable 1 (*P_exp,T_* (kN))	Variable 2 (P_u(Wu)_ (kN))
Mean	34.608	14.332
Variance	100.729	0.043
Observations	15	15
Hypothesized Mean Difference	0	
df	14	
t Stat	7.822	
*P* (T ≤ t) one-tail	8.882 × 10^−7^	
t Critical one-tail	1.7613	
*P* (T ≤ t) two-tail	1.776 × 10^−6^	
t Critical two-tail	2.145	

## Data Availability

The original contributions presented in this study are included in the article. Further inquiries can be directed to the corresponding authors.

## Abbreviations

The following abbreviations are used in this manuscript:

CFRP	Carbon fiber-reinforced polymer
RC	Reinforced concrete
LVDT	Linear variable differential transducer
$P_{max,T}$	Bond strength at temperature of T °C
$P_{max,20}$	Bond strength at room temperature of 20 °C
$\alpha_{1}$	The parameter considering the effect of temperature
$\alpha_{2}$	The parameter considering the effect of the duration of exposure to fire
$\alpha_{3}$	The parameter considering the effect of the method of cooling/extinguishing
$\beta_{p}$	The coefficient variable influence of the pasting width of the CFRP sheets
$\beta_{L}$	The coefficient variable influence of the pasting length of the CFRP sheets
$L_{eT}$	Effective bond length
∆	The parameter calculated using a regression analysis of the predicted and experimental bond strength between fire-damaged concrete and CFRP sheets
T	Temperature
t	The duration of exposure to fire
$f_{cT}^{\prime }$	The compression strength of concrete at any temperature
$b_{p}$	The width of the CFRP paste
FRP	Fiber-reinforced polymer
$b_{c}$	The width of the concrete specimen
L	The bonding length of CFRP
L_e_	The effective or the active bonding length between CFRP and concrete, which is the length over which most of the bond stress is maintained (ACI 440.2R-23)
$E_{f}$	Young’s modulus of carbon fiber
$n_{f}$	The number of layers of carbon fiber
$t_{f}$	The thickness of carbon fiber
COV	Coefficient of variation

## References

[1] Mostafa M.M.A. (2024). Numerical FE Modeling and Design Methods of CCES Columns with Normal-Weight Crushed Dolomite Coarse Aggregate Fully Embedded IPE Steel-Section. Int. J. Concr. Struct. Mater..

[2] Mostafa M.M.A. (2023). Analysis and design of eccentrically loaded lightweight aggregate concrete-encased steel slender columns. Struct. Eng. Mech..

[3] Megahed F.A., Seleem M.H., Badawy A.A.M., Sharaky I.A. (2023). The flexural response of RC beams strengthened by EB/NSM techniques using FRP and metal materials: A state-of-the-art review. Innov. Infrastruct. Solut..

[4] Mostafa M.M.A., Wu T., Liu X., Fu B. (2021). Axial behavior of the steel reinforced lightweight aggregate concrete (SRLAC) short columns. Steel. Comp. Struc..

[5] Elamary A.S., Sharaky I.A., Alharthi F.M., El-Zohairy A., Mostafa M.M.A. (2025). The Influence of Various Tensile and Shear Reinforcement Configurations on the Ultimate Capacity and Failure Mechanisms of Reinforced Concrete Beams. Buildings.

[6] Wang J.H., Zhang X., Kunnath S., He J., Xiao Y. (2021). Post-Earthquake Fire Resistance and Residual Seismic Capacity of Reinforced Concrete Columns. ACI Struct. J..

[7] Kodur V.K. (2018). Innovative strategies for enhancing fire performance of high-strength concrete structures. Adv. Struct. Eng..

[8] Wang W., Han L.-H., Tan Q., Tao Z. (2017). Tests on the steel–concrete bond strength in steel reinforced concrete (SRC) columns after fire exposure. Fire Technol..

[9] Shakib H., Pirizadeh M., Dardaei S., Zakersalehi M. (2018). Technical and administrative assessment of Plasco building incident. Int. J. Civ. Eng..

[10] Liu P., Zhou X., Qian Q., Berto F., Zhou L. (2019). Dynamic splitting tensile properties of concrete and cement mortar. Fatigue Fract. Eng. Mater. Struct..

[11] Phng E.G.H. (2006). Fire Resistance of Steel and Composite Columns. Master’s Thesis.

[12] Lubl’oy E. (2021). Fire Resistance of Concrete Based Structures.

[13] Schrefler B.A., Brunello P., Gawin D., Majorana C.E., Pesavento F. (2002). Concrete at high temperature with application to tunnel fire. Comput. Mech..

[14] Annerel E., Taerwe L. (2009). Revealing the temperature history in concrete after fire exposure by microscopic analysis. Cem. Concr. Res..

[15] Lu X., Lu X., Guan H., Ye L. (2012). Collapse simulation of reinforced concrete high-rise building induced by extreme earthquakes. Earthq. Eng. Struct. Dyn..

[16] Khoury G.A. (2000). Effect of fire on concrete and concrete structures. Prog. Struct. Eng. Mater..

[17] Tomar M.S., Khurana S. (2019). Impact of passive fire protection on heat release rates in road tunnel fire: A review. Tunn. Undergr. Space Technol..

[18] Zhao Y., Bi J., Zhou X., Huang Y. (2019). Effect of High Temperature and High Pressure of Water on Micro-Characteristic and Splitting Tensile Strength of Gritstone. Front. Earth Sci..

[19] Du S., Zhang Y., Sun Q., Gong W., Geng J., Zhang K. (2018). Experimental study on color change and compression strength of concrete tunnel lining in a fire. Tunn. Undergr. Space Technol..

[20] Schneider U., Lebeda C. (2000). Fire Protection of Buildings. (Baulicher Brandschutz).

[21] Schneider U., Horvath J. (2003). Behaviour of Ordinary Concrete at High Temperatures.

[22] Botte W., Caspeele R. (2017). Post-cooling properties of concrete exposed to fire. Fire Saf. J..

[23] Jian Y. (2004). Research Advance in FRP strengthened RC structures. Bull. Sci. Technol..

[24] Hosen M.L., Raju M.K.B., Mahmud H.M.I. (2021). The Effect of Fire on the Strength of Concrete Material. J. Eng. Sci..

[25] Yang W., Yang Y., Liu F., Chen Y.F. (2023). Fire experiment and fire design method of U-shaped steel-concrete composite beams. Adv. Struct. Eng..

[26] Yang Y., Chang H., Wang C. (2021). Study on post-fire performance and residual capacity of steel reinforced high-strength concrete composite columns under eccentric compression. IOP Conf. Ser. Earth Environ. Sci..

[27] Tan Q.-H., Gardner L., Han L.-H., Song T.-Y. (2020). Performance of concrete-filled stainless-steel tubular (CFSST) columns after exposure to fire. Thin Walled Struct..

[28] Li Q.-H., Sun C.-J., Lyu J.-F., Quan G., Huang B.-T., Xu S.-L. (2020). Fire Performance of Steel-Reinforced Ultrahigh-Toughness Cementitious Composite Columns: Experimental Investigation and Numerical Analyses. J. Struct. Eng..

[29] Han L.-H., Zhou K., Tan Q.-H., Song T.-Y. (2020). Performance of steel reinforced concrete columns after exposure to fire: Numerical analysis and application. Eng. Struct..

[30] Wu T., Sun Y., Liu X., Wei H. (2019). Flexural Behavior of Steel Fiber–Reinforced Lightweight Aggregate Concrete Beams Reinforced with Glass Fiber–Reinforced Polymer Bars. J. Compos. Constr..

[31] Chen G., Wang Y., Yu T., Zhang B., Han B. (2022). Elliptical FRP–Concrete–Steel Double-Skin Tubular Columns: Axial Behavior, Interaction Mechanism, and Modeling. J. Compos. Constr..

[32] Cheng S., Feng P., Bai Y., Ye L.P. (2016). Load-Strain Model for Steel-Concrete-FRP-Concrete Columns in Axial Compression. J. Compos. Constr..

[33] El-Maaddawy T., El-Dieb A.S. (2010). Near-surface-mounted composite system for repair and strengthening of reinforced concrete columns subjected to axial load and biaxial bending. J. Compos. Constr..

[34] Pham T.M., Hadi M.N., Youssef J. (2015). Optimized FRP wrapping schemes for circular concrete columns under axial compression. J. Compos. Constr..

[35] Razaqpur A.G., Spadea S. (2015). Shear Strength of FRP Reinforced Concrete Members with Stirrups. J. Compos. Constr..

[36] Rajak D.K., Pagar D.D., Menezes P.L., Linul E. (2019). Fiber-Reinforced Polymer Composites: Manufacturing, Properties, and Applications. Polymers.

[37] Parvin A., Altay S., Yalcin C., Kaya O. (2010). CFRP Rehabilitation of Concrete Frame Joints with Inadequate Shear and Anchorage Details. J. Compos. Constr..

[38] Xu Z.-s., Guo X.-w., Zhang Y. (2005). Experimental on Using CFRP to Strengthen Full Scale Reinforced Concrete Beam after Fire. Fire Saf. Sci..

[39] Zu X., Feng K., Zhang W., Yang Z. (2005). Experimental Analysis of CFRP Used to Strengthen RC Beam After Fire. J. Harbin Inst. Technol..

[40] Xiang K., Wang G., Zhao T., Lu Z. (2010). Experimental Research on Carbon Fiber Reinforced Polymer Sheets Strengthened Fire-damaged Reinforced Continuous Beams. J. Sichuan Univ. Eng. Sci. Ed..

[41] Bisby L.A., Chen J., Li S., Stratford T., Cueva N., Crossling K. (2011). Strengthening Fire Damaged Circular Concrete Columns with FRP. Advanced Composites in Construction.

[42] Abdel-Hafez L.M., Abouelezz A.E.Y., Hassan A.M. (2014). Behavior of RC columns retrofitted with CFRP exposed to fire under axial load. HBRC J..

[43] Wang L.-G., Lu Z.-D., Sheng Q.-Z. (2003). Experimental Research Concrete Frame After High Temperature Rehabilitated by CFRP. Fire Saf. Sci..

[44] Haddad R.H., Al-Rousan R., Almasry A. (2013). Bond-slip behavior between carbon fiber reinforced polymer sheets and heat-damaged concrete. Compos. Part B Eng..

[45] Xu Y., Lin Y., Yang Q., Lin B.-l. (2014). Experimental study on seismic performance of concrete short columns after fire and strengthened with CFRP. Eng. Mech..

[46] Liu G., Qu F., Zhao S., Zhang H. (2019). Bond-slip behavior between CFRP sheets and heat-damaged concrete. J. Build. Struct..

[47] Al-Rousan R., Al-Tahat M. (2020). An Anchoring Groove Technique to Enhance the Bond Behavior between Heat-Damaged Concrete and CFRP Composites. Buildings.

[48] Fehérvári S. (2022). Effect of Cooling Methods on the Residual Properties of Concrete Exposed to Elevated Temperature. Results Eng..

[49] Zhao K., Hu Z., Wang B., Wen Y., Han J., Wu Q., Xu Y. (2024). Experimental investigation on axial compression behavior of heat-damaged concrete cylinders confined with CFRP sheets. Structures.

[50] Madani S.A., Hatami S., Farahbod F., Jamali Ashtiani M. (2024). FRP strip wraps for enhancing the fire resistance of RC beams strengthened with CFRP sheets bonded using the EBROG method: An experimental study. Structures.

[51] Dong K., Hu K.-X., Gao W.-Y., Yang S.-T. (2023). Fire endurance tests of CFRP-strengthened RC beams with different insulation schemes. Structures.

[52] Obaidat Y.T., Barham W., Obeidat Y.H. (2024). Repairing thermally shocked reinforced concrete beams using innovative CFRP sheets anchorage systems. Structures.

[53] Wu F., Lei S., Liu Z., Cao J., Liu L. (2024). Flexural behavior of RC beams strengthened with CFRP grid-reinforced engineering cementitious composite. Structures.

[54] Alkhateeb M.Y., Hejazi F. (2022). Reinforced concrete beams externally strengthened by CFRP rods with steel plate, anchorage bolts, and concrete jacketing. Structures.

[55] Akbarzadeh Bengar H., Hosseinpour M., Celikag M. (2020). Influence of CFRP confinement on bond behavior of steel deformed bar embedded in concrete exposed to high temperature. Structures.

[56] Al-Rousan R.Z., Al-Tahat M.F. (2020). Consequence of anchoring holes technique on the bond behavior between CFRP composites and heat-damaged concrete. Structures.

[57] Abadel A.A., Masmoudi R., Iqbal Khan M. (2022). Axial behavior of square and circular concrete columns confined with CFRP sheets under elevated temperatures: Comparison with welded-wire mesh steel confinement. Structures.

[58] Haris M., Xiong E., Gao W., Samuel M.A., Sahar N.U., Saleem A. (2024). Strengthening Reinforced Concrete Members Using FRP-Evaluating Fire Performance, Challenges, and Future Research Directions: A State-of-the-Art Review. Polymers.

[59] Seo S.-y., Lim J.-w., Jeong S.-h. (2021). Evaluation on the Bond Capacity of the Fire-Protected FRP Bonded to Concrete Under High Temperature. Int. J. Concr. Struct. Mater..

[60] Mar Swe T., Limpaninlachat P., Daungwilailuk T., Pansuk W., Pheinsusom P. (2023). Bonding behavior of interface between reinforced concrete after fire and carbon fiber-reinforced polymer. Constr. Build. Mater..

[61] Ahmed A., Kodur V.K.R. (2011). Effect of bond degradation on fire resistance of FRP-strengthened reinforced concrete beams. Compos. Part B Eng..

[62] Al-Rousan R.Z., Alnemrawi B.a.R. (2025). Empirical and precise finite element modelling of bond-slip contact behavior between heat-damaged concrete and anchored CFRP composites with groove. Eng. Struct..

[63] Thongchom C., Lenwari A., Aboutaha R.S. (2020). Bond Properties between Carbon Fibre-Reinforced Polymer Plate and Fire-Damaged Concrete. Int. J. Adhes. Adhes..

[64] Ombres L., Mazzuca P., Micieli A., Candamano S., Campolongo F. (2025). FRCM–Masonry Joints at High Temperature: Residual Bond Capacity. J. Mater. Civ. Eng..

[65] (2019). Standard for Test Methods of Concrete Physical and Mechanical Properties.

[66] (2017). Standard Test Method for Tensile Properties of Polymer Matrix Composite Materials.

[67] (2017). Standard Test Method for Determining Tensil Properties of Fiber Reinforced Polymer Matrix Composites Used for Strengthening of Civil Structures.

[68] Alsuhaibani E., Yazdani N., Beneberu E. (2022). Durability and Long-Term Performance Prediction of Carbon Fiber Reinforced Polymer Laminates. Polymers.

[69] Raheem S.A. (2015). The Effect of Recycled Heating and Cooling and The Effect of The Specimen Size on The Compressive Strength of Concrete Exposed to High Temperature. J. Eng..

[70] (2008). Design Handbook for RC Structures Retrofitted with FRP and Metal Plates: Beams and Slabs.

[71] Alhassan M.A., Al Rousan R.Z., Al Shuqari E.A. (2019). Bond-slip behavior between fiber reinforced concrete and CFRP composites. Ain Shams Eng. J..

[72] Chen C., Li X., Wang X., Sui L., Xing F., Li D., Zhou Y. (2019). Effect of transverse groove on bond behavior of FRP-concrete interface: Experimental study, image analysis and design. Compos. Part B: Eng..

[73] Al-Rousan R.Z. (2018). Empirical and NLFEA prediction of bond-slip behavior between DSSF concrete and anchored CFRP composites. Constr. Build. Mater..

[74] Al-Rousan R.Z., Al-Tahat M.F. (2019). Consequence of surface preparation techniques on the bond behavior between concrete and CFRP composites. Constr. Build. Mater..

[75] Karakoç M.B. (2013). Effect of cooling regimes on compressive strength of concrete with lightweight aggregate exposed to high temperature. Constr. Build. Mater..

[76] Zaidi K.A., Sharma U.K., Bhandari N.M. (2012). Effect of temperature on uni-axial compressive behavior of confined concrete. Fire Saf. J..

[77] Yüzer N., Aköz F., Öztürk L.D. (2004). Compressive strength–color change relation in mortars at high temperature. Cem. Concr. Res..

[78] Wang Z., Dai J.-G., Wang M., Chen L., Zhang F., Xu Q. (2021). Residual Bond Strengths of Epoxy and Cement-Bonded CFRP Reinforcements to Concrete Interfaces after Elevated Temperature Exposure. Fire Saf. J..

[79] Guo X., Xie K., Liu Z., Elchalakani M., Zhou Y. (2023). A Study on the Bond Strength of Interface between Post-Fire Concrete and Different Epoxy Resin Adhesives. Constr. Build. Mater..

[80] Liu G., Wang Y., Qu F., Guo X., Li Y., Cheng S. (2023). Bonded Behavior of Hybrid-Bonded CFRP to Heat-Damaged Concrete Interface. Buildings.

[81] Andrade M.S.A., Ribeiro J.C.L., Oliveira D.S.d., Pedroti L.G., Santos C.F.R. (2024). Experimental evaluation of concrete-reinforcement bond: Bond failure mechanisms after exposure to elevated temperatures. Eng. Struct..

[82] Al-barawi A. (2021). Experimental Study of Bond Behavior Between CFRP Sheet and Fire Damaged Concrete. Master’s Thesis.

[83] Chen J.F., Teng J.G. (2001). Anchorage Strength Models for FRP and Steel Plates Bonded to Concrete. J. Struct. Eng..

[84] (2023). Design and Construction of Externally Bonded Fiber-Reinforced Polymer (FRP) Systems for Strengthening Concrete Structures—Guide.

[85] Wu Z., Islam S.M., Said H. (2008). A Three-Parameter Bond Strength Model for FRP—Concrete Interface. J. Reinf. Plast. Compos..

